# Effects of Subthalamic and Nigral Stimulation on Gait Kinematics in Parkinson’s Disease

**DOI:** 10.3389/fneur.2017.00543

**Published:** 2017-10-17

**Authors:** Marlieke Scholten, Johannes Klemt, Melanie Heilbronn, Christian Plewnia, Bastiaan R. Bloem, Friedemann Bunjes, Rejko Krüger, Alireza Gharabaghi, Daniel Weiss

**Affiliations:** ^1^Department of Neurodegenerative Diseases and Hertie Institute for Clinical Brain Research (HIH), University of Tuebingen, Tuebingen, Germany; ^2^German Centre of Neurodegenerative Diseases (DZNE), University of Tuebingen, Tuebingen, Germany; ^3^Centre for Integrative Neuroscience (CIN), University of Tuebingen, Tuebingen, Germany; ^4^Graduate School of Neural & Behavioural Sciences, International Max Planck Research School, University of Tuebingen, Tuebingen, Germany; ^5^Department of Psychiatry and Psychotherapy, University of Tuebingen, Tuebingen, Germany; ^6^Radboud University Medical Centre, Department of Neurology, Donders Institute for Brain, Cognition and Behavior, Nijmegen, Netherlands; ^7^Clinical and Experimental Neuroscience, Luxembourg Centre for Systems Biomedicine (LCSB), University of Luxembourg Center Hospitalier de Luxembourg (CHL), Luxembourg, Luxembourg; ^8^Division of Functional and Restorative Neurosurgery, Department of Neurosurgery, University of Tuebingen, Tuebingen, Germany

**Keywords:** Parkinson’s disease, subthalamic nucleus deep brain stimulation, gait, freezing of gait, nigral stimulation

## Abstract

Conventional subthalamic deep brain stimulation for Parkinson’s disease (PD) presumably modulates the spatial component of gait. However, temporal dysregulation of gait is one of the factors that is tightly associated with freezing of gait (FOG). Temporal locomotor integration may be modulated differentially at distinct levels of the basal ganglia. Owing to its specific descending brainstem projections, stimulation of the substantia nigra pars reticulata (SNr) area might modulate spatial and temporal parameters of gait differentially compared to standard subthalamic nucleus (STN) stimulation. Here, we aimed to characterize the differential effect of STN or SNr stimulation on kinematic gait parameters. We analyzed biomechanical parameters during unconstrained over ground walking in 12 PD patients with subthalamic deep brain stimulation and FOG. Patients performed walking in three therapeutic conditions: (i) Off stimulation, (ii) STN stimulation (alone), and (iii) SNr stimulation (alone). SNr stimulation was achieved by stimulating the most caudal contact of the electrode. We recorded gait using three sensors (each containing a tri-axial accelerometer, gyroscope, and magnetometer) attached on both left and right ankle, and to the lumbar spine. STN stimulation improved both the spatial features (stride length, stride length variability) and the temporal parameters of gait. SNr stimulation improved temporal parameters of gait (swing time asymmetry). Correlation analysis suggested that patients with more medial localization of the SNr contact associated with a stronger regularization of gait. These results suggest that SNr stimulation might support temporal regularization of gait integration.

## Introduction

Standard deep brain stimulation of the subthalamic nucleus (STN-DBS) may improve gait in Parkinson’s disease (PD) with interindividual variability (1, 2). STN-DBS was shown to modulate the spatial- and spatiotemporal parameters (e.g., step length, step velocity), however, the pure temporal parameters (e.g., step time) were less amenable to STN-DBS (3–5) and possibly more dependent on contributions from the mesencephalic locomotor region (MLR) along previous findings (6, 7). Taken together, the spatial and temporal parameters of gait do not act independently; however, there may be weighted contributions to either spatial or temporal parameters from different nodes of the wide-spread locomotor network. This is critical, as temporal regulation of the gait cycle is crucial to PD gait disturbance, in particular to PD patients with freezing of gait (FOG). Temporal abnormalities of locomotor integration may increase susceptibility to FOG (8). In this sense, several temporal parameters of gait, including temporal gait variability and asymmetry, are deteriorated in PD freezers (9, 10).

Rhythmic stepping behavior can be elicited by stimulation of the MLR in cat, with higher stimulation intensities increasing the cadence along temporal locomotor integration (11, 12). The MLR gives rise to the reticulospinal tract and this tract appears to be involved in eliminating asymmetric gait by modulating the activity level of different groups of muscles during walking (13). In human beings, the MLR was referred to the pedunculopontine and cuneiform nuclei in previous work (6, 12, 13). The MLR appears to be involved in the temporal modulation of gait in human beings as well (6, 7). The MLR appears to be involved in the temporal modulation of gait in human as well. In healthy subjects, the MLR is active during mental imagery of gait (6). Furthermore, increased cadence of stepping in PD patients associates with increased firing rates of neurons in the MLR (7). The MLR receives GABAergic afferents from the substantia nigra pars reticulata (SNr), a major basal ganglia output nucleus (14). In PD, the output of the basal ganglia including SNr is increased (15), presumably resulting in over-inhibition of the MLR. Accordingly, increased inhibitory SNr output is suspected to attenuate MLR locomotor output.

High-frequency stimulation likely attenuates over-inhibitory output both on the level of STN and SNr (16, 17). There is clinical evidence that STN-DBS modulates locomotor integration and FOG (18, 19). A recent trial observed that adding nigral stimulation to the conventional STN-DBS may improve gait in PD freezers (20). Efficacy of STN + SNr on FOG as primary clinical outcome measure is under investigation in a multicenter randomized controlled trial (clinicaltrials.gov NCT02588144).

Here, we set out to study the effects of mono STN or mono SNr stimulation (each applied as single target stimulation) on gait kinematics of PD freezers. We conducted this study under the primary interest, whether mono STN or mono SNr stimulation would elicit differential effects on locomotion in terms of kinematic gait properties. Of note, this pathophysiological groundwork here was not designed nor powered to study the clinical efficacy of nigral stimulation on FOG. In particular, we contrasted the effect of either STN or SNr stimulation as single target stimulation on both spatial and temporal kinematic gait parameters of unconstrained gait, which would provide insight into the underlying pathophysiological and network effects of each distinct target within the complex locomotor network. Furthermore, we aimed to associate the anatomical position of SNr contacts with their potential to modulate gait parameters, since—from animal experiments—it was indicated that SNr subterritories may differentially modulate distinct parameters of locomotor integration. In this sense, the medial part of the SNr was suggested to account for the modulation of the cadence (21, 22). Instead, stimulation of the lateral part of the SNr presumably led to an increase of axial and limbic muscle tone (21, 23). We hypothesize that STN stimulation will modulate the spatial parameters by increasing the stride length and reducing the stride length variability as compared to Off stimulation. We hypothesize that SNr stimulation (owing to its MLR connectivity) regularizes the temporal measures of gait as compared to Off stimulation by decreasing the variability of the step time and temporal gait asymmetry. Furthermore, we hypothesize that the improvement in temporal gait variability and asymmetry may associate with a more medial localization of the SNr contact.

## Materials and Methods

### Subjects

We included 21 patients with idiopathic PD and STN-DBS into this study. PD patients were included in the post-operative chronic follow-up period, if already pre-existing electrode contacts of the quadripolar lead (electrode model 3389, Medtronic, Minneapolis, MN, USA) reached both the STN and the caudal border zone of the STN and SNr (20). The pre-operative targeting occurred under routine clinical care (not subject to this study protocol) and primarily focused on the STN without specifically targeting the SNr. However, for patient inclusion into this study, we identified from the available post-operative images whether the lowermost contact was located at least −5 mm below the mid-commissural point (MCP) in the rostro-caudal direction. This resembled the standards of our randomized controlled clinical trial on STN + SNr stimulation owing to the natural variability of electrode depth which may reach below the STN toward SNr or STN–SNr border zone (clinicaltrials.gov NCT02588144).

To avoid influence of the stun effect, we included only patients at least 3 months from STN-DBS implantation. Exclusion criteria were Mini Mental Status <22, Beck’s Depression Inventory >13, and other neurological or neuromuscular disease except PD. The local Ethics committee of the University of Tuebingen approved the study and all subjects provided written consent to participate in the study.

We excluded one patient because of technical problems with the recording hardware and one patient because he was unable to walk during the recording session. We observed FOG in 15 out of 19 PD patients, confirming these PD patients as “definitive freezers” (24). FOG was triggered by 360° narrow turns in both directions in the “Off” state before the measurement. This task can trigger FOG most effectively (24). We assured that stimulation of the STN was effective with clinical improvement of at least 30% measured as Unified Parkinson’s Disease Rating Scale part 3 (UPDRS III) difference between Off stimulation and STN stimulation. Thus, we excluded 3 out of 15 PD patients.

We considered the remaining 12 PD freezers for further analyses [PD1–PD12, 11 males, age: 63.7 ± 10.4 years (mean ± SD)]. The disease duration was 15.1 ± 3.2 years with implantation of the DBS 34.7 ± 29.0 months ago. The daily levodopa equivalent dose was on average 483 ± 327 mg. The score on the NFOG-Q was 9.4 ± 8.4 points. Pre-operatively, patient records pointed to 10 out of 12 with FOG. Electrode coordinates of the caudal contact relative to the MCP were left SNr −10.2 ± 0.4, −3.7 ± 0.5, −6.5 ± 0.4; right SNr 10.4 ± 0.4, −4.0 ± 0.4, −6.3 ± 0.4 (x, y, z; x, medio-lateral; y, anterio-posterior; z, rostro-caudal).

### Experimental Setup and Paradigm

Combined STN + SNr stimulation is under consideration to alleviate FOG (20). Whereas in current clinical trials impulses of STN and SNr are delivered concomitantly, we aimed to gain differentiated pathophysiological insight of either STN or SNr stimulation as single target stimulation on locomotor integration in this study. To this end, we contrasted the effects of either stimulation condition alone (i.e., mono SNr as compared to mono STN). We measured all patients in three conditions: STN, SNr, and Off stimulation after overnight withdrawal of dopaminergic medication. As a result, all measurements were performed “Off medication.” We assigned the order of the conditions randomly. Experimenters and patients were not blinded to the stimulation condition. Each stimulation condition was active for at least 20 min prior to the recordings in order to achieve sufficient efficacy of stimulation and in order to limit potential carry-over effects of the previous therapeutic condition in line with previous standards (20, 25). As recordings took place in medication off, we did not consider longer off periods for washout, which would have led to patient discomfort.

Subthalamic nucleus stimulation represented stimulation of the rostral contact(s), while we delivered SNr stimulation on the caudal contact(s) (Table 1). Pulse width and frequency of the SNr stimulation were similar to the individual STN stimulation. We did not adjust SNr to STN amplitudes, since stimulation of nigral contacts (i) generally has a lower threshold for capsular side effects and (ii) lower SNr amplitudes (as compared to STN amplitudes) were clinically effective in our previous work (20). In this sense, we designed the stimulation conditions close to current clinical reprogramming standards on subthalamic and nigral stimulation. Therefore, the voltage was increased in small steps of 0.1 V until a clinical improvement of gait compared to Off stimulation was observed. If a side effect occurred, the highest amplitude possible without the emergence of side effects was chosen (Table 1). On average, we stimulated the left STN with 3.8 ± 1.3 V and the right STN with 3.0 ± 1.1 V. The left SNr was on average stimulated with 1.9 ± 1.0 V and the right SNr with 1.9 ± 0.9 V. Information on the potential side effects of SNr stimulation were reported in detail elsewhere (20). In this study, we were limited in amplitude increases on nigral contacts only in one patient (PD11), who showed blurred vision around amplitudes of 0.5 V and, therefore, was treated with rather low amplitudes in the SNr of bilateral 0.3 V (Table 1). In each condition, subjects walked on a straight over ground walkway of 9 m for kinematic gait analysis. Patients walked for about 3 min in their self-selected, comfortable pace. In case the PD patients had difficulty walking 3 min, they were asked to walk as long as they could. Before the start of each 9-m walking trajectory, subjects stood still for a few seconds with the feet put together in front of the starting line. Walking was self-initiated by the subjects. Walking aid, such as a cane or walking frame was allowed when used in daily life. The walking aid was used by only two patients and consistently used in all therapeutic conditions to facilitate comparability. In each condition, we assessed the clinical motor state using the UPDRS section III. Clinical subscores were composed: segmental (sum of items 20–26 + 31, only upper and lower limbs) and the gait and posture subscores (sum of items 27–30).

**Table 1:** Stimulation parameters.

	STN				SNr			
	Voltage (V) (left/right)	Contacts (left/right)	Frequency (Hz)	Pulse width (μs) (left/right)	Voltage (V) (left/right)	Contacts (left/right)	Frequency (Hz)	Pulse width (μs) (left/right)
PD01	5.3/3.0	2–3+/10–11+	125	60/60	3.5/3.5	0–1+/8–9+	125	60/60
PD02	2.8/3.5	2–3+/10–11+	130	60/60	2.5/1.9	0–1+/8–9+	130	60/60
PD03	5.5/3.5	2–3+/10–11+	130	60/60	2.9/2.9	0–1+/8–9+	130	60/60
PD04	4.0/4.5	2–3+/10–11+	130	60/60	2.7/2.7	0–1+/8–9+	130	60/60
PD05	2.1/2.1	2–3+/10–11+	125	60/60	1.6/1.6	0–1+/8–9+	125	60/60
PD06	3.2/2.0	2–3+/10–11+	130	60/60	2.2/2.2	0–1+/8–9+	130	60/60
PD07	5.4/5.1	2–3+/10–11+	130	90/90	1.3/1.3	0–1+/8–9+	130	90/90
PD08	4.9/3.5	1-C+/9-C+	130	90/60	2.5/2.5	0-C+/8-C+	130	90/60
PD09	3.5/2.4	2-C+/10-C+	125	60/60	1.6/1.6	0-C+/8-C+	125	60/60
PD10	1.8/1.7	2-C+/10-C+	125	90/90	1.0/1.0	0-C+/8-C+	125	90/90
PD11	3.6/1.9	2–3+/10–11+	130	60/60	0.3/0.3	0–C+/8-C+	130	60/60
PD12	3.8/2.4	2–3+/10–11+	125	60/60	0.8/0.8	0-C+/8-C+	125	60/60

*STN, subthalamic nucleus deep brain stimulation; SNr, substantia nigra deep brain stimulation*.

*Remark: after exclusion of PD11, only the gait asymmetry between SNr and Off stimulation did not reach significance anymore*.

### Recordings

During walking, participants wore small, lightweight body-fixed sensors attached to the left and right ankle (about 20 mm above the malleolus), and to the lumbar spine with a belt (Opal, APDM, Portland, OR, USA). Each sensor contained a tri-axial accelerometer, tri-axial gyroscope, and a tri-axial magnetometer with *X, Y*, and *Z* axes pointing in subjects’ viewing direction downward, rightward, and forward, respectively. Data were sampled at 128 Hz and transferred to Matlab (Release R2015b, The Mathworks, Inc., Natick, MA, USA) for further offline analysis. Furthermore, the participants were video-taped to exclude dyskinesias and FOG episodes from analysis.

### Spatial and Temporal Gait Parameters

We obtained gait events from the recorded inertial signals based on earlier work showing a good agreement between APDM sensors and the GAITRite system as gold standard (26, 27). Moreover, Opal sensors of APDM are well established and widely used. Gait events were determined using the acceleration in the anterior–posterior direction and the gyroscope in the medial–lateral direction expressing the angular velocity in the sagittal plane. Briefly, first we identified the midswing (MS) as peak value exceeding 50°/s in the sagittal plane of the gyroscope signal. If multiple peak values with a maximum distance of 750 ms were found, the highest peak was selected and the others were rejected. In the time interval 750 ms before and after MS, we identified toe-off (TO) and heel-strike (HS). TO was identified as minimum anterior–posterior acceleration in the time interval before MS, and HS was identified in the time interval after MS as the minimum value of angular velocity in the sagittal plane before the maximum anterior–posterior acceleration. A stride was defined as the time span between two consecutive HS of the same leg. All gait cycles were checked for the order of occurrence. For the *k*th gait cycle of the left leg the gait events were correct when:

HS_L_ < TO_R_ < MS_R_ < HS_R_ < TO_L_ < MS_L_ < HS_L_.

We discarded gait cycles from further analyses in case of incorrect order of the gait events or if a gait event could not be detected. To exclude acceleration and deceleration during walking, the first and last two steps of each 9 m walkway were rejected. A minimal of 40 gait cycles (for a particular leg) remained for each participant in each condition for further analyses.

Using the gait events, we computed temporal and spatial gait outcome measures for each condition. We used as temporal measures the mean step time (in seconds, time between HS of one leg and HS of the other leg) and the variability of the step time. As spatial measures, we computed the stride length [as percentage of the leg length (% ll)] and the variability of stride length. Spatiotemporal consisted of the peak shank angular velocity (degree/seconds; sagittal maximum angular velocity during the swing phase) and the variability of peak shank angular velocity. The variability measures were calculated as the coefficient of variation (CV, 100%*SD normalized by the mean). We report the measures grouped by the more severely affected leg and less severely affected leg by PD (referred to as disease “dominant leg” resp. “non-dominant leg” in the following). As most objective marker, the dominant leg was determined as the leg yielding lower peak angular velocity during the swing phase in the Off condition as compared to the other leg.

Freezers have impaired bilateral coordination (28). Therefore, we observe the swing time asymmetry (STA) of the swing time defined in Ref. (29).

STA=|ln(lh)|,

where *l* is the swing time of the leg with the shorter swing time and *h* is the swing time of the leg with the greater swing time. STA closer to 0 represents a lower grade of asymmetry.

### Anatomical Position of SNr Contact

We determined the electrode position with respect to MCP of the bilateral lower contacts using co-registration of the pre-operative and post-operative MRI with Optivise software (Medtronic, Minneapolis, MN, USA). As previous animal work points to the relevance of laterality within the SNr regarding the modulation of locomotor integration (22), we aim to further explore the role of electrode positioning. Therefore, we correlated the medio-lateral coordinate of the electrode (coordinate in *x* direction) with the spatial and temporal parameters of the dominant leg and STA.

### Statistical Analyses

All statistical analyses were performed with exploratory intent. We report descriptive statistics as mean ± SD, unless stated otherwise. We tested for normal distribution of the data using the Kolmogorov–Smirnov test (*p* < 0.05). Based on this test, conditions were compared using a repeated measure ANOVA or Friedman test. In case of a significant ANOVA or Friedman test, *post hoc* tests were performed comparing Off with STN and Off with SNr stimulation, respectively, using a paired *t*-test or Wilcoxon test. All statistical analyses were performed with IBM SPSS statistics, version 22.0 (IBM Deutschland GmbH, Ehningen, Germany). Since one patient (PD11) could only be treated with reduced SNr amplitudes of 0.3 V owing to side effects in terms of blurred vision at higher amplitudes, we reanalyzed findings from SNr stimulation after exclusion of PD11.

## Results

### Clinical Outcome

**Figure 1:**
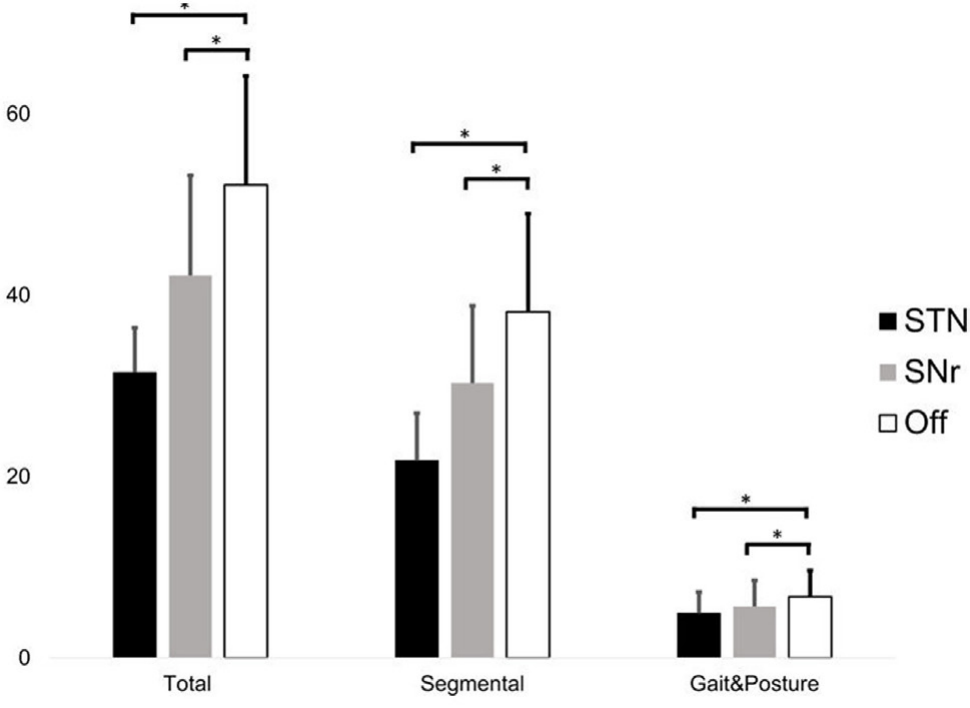
Score of the total UPDRS III (left), segmental score (sum of items 20–26 + 31, only upper and lower limbs), and gait and posture subscore (sum of items 27–30) during subthalamic nucleus (STN), substantia nigra pars reticulate (SNr), and Off stimulation. Significant differences (*p* < 0.05) are denoted by horizontal square brackets. Both STN and SNr stimulations could significantly improve the segmental and gait and posture subscore.

Total UPDRSIII score significantly differed between conditions (χ^2^ = 22.167, *p* < 0.001, Friedman test). STN stimulation improved the total UPDRSIII score with 38 ± 13% (percentage improvement of STN stimulation compared to “Off” stimulation, normalized by the score in Off stimulation, STN–Off *U* = −3.059, *p* = 0.002) and SNr stimulation with 18 ± 16% (SNr–Off *U* = −2.671, *p* = 0.008, Wilcoxon Test, Figure 1). The segmental subscore (χ^2^ = 22.167, *p* < 0.001) and the gait and posture subscore (χ^2^ = 15.235, *p* < 0.001) were changed by stimulation. STN stimulation improved the segmental subscore by 41 ± 13% and SNr stimulation by 19 ± 17% (STN–Off *U* = −3.061, *p* = 0.002; SNr–Off *U* = −2.515, *p* = 0.012). STN stimulation improved the gait and posture subscore by 27 ± 14% and SNr stimulation by 18 ± 20% (STN–Off *U* = −2.980, *p* = 0.003; SNr–Off *U* = −2.280, *p* = 0.023) (Figure 1). All findings of SNr–Off stimulation on the UPDRSIII score and UPDRS III subscores remained after exclusion of PD11 (all *p* < 0.05).

### Spatial and Temporal Gait Parameters

Spatial parameters were only influenced by STN stimulation: we observed a stride length increment and stride length variability reduction compared to Off stimulation for the dominant leg (Table 2). SNr stimulation did not change the spatial parameters compared to Off stimulation. The variability of the step time was decreased by STN stimulation for both the dominant and non-dominant leg (Table 2). The step time and both average and variability of the peak shank angular velocity were not influenced by both STN and SNr stimulations.

**Table 2:** Dominant/non-dominant leg: spatial and temporal gait parameters.

Gait parameter	Grand average (mean ± SD)						*p*-Value		*p*-Value	
	
	STN		SNr		Off		STN–Off		SNr–Off	
			
	D	ND	D	ND	D	ND	D	ND	D	ND
**Mean**										
Stride length (% ll)	66.1 ± 25.5	74.5 ± 21.7	54.8 ± 27.2	65.2 ± 26.8	53.1 ± 28.5	63.4 ± 27.8	**0.01**	n.s.	0.27	n.s.
PAV (deg/s)	226.3 ± 83.4	260.7 ± 77.4	202.2 ± 87.2	238.0 ± 76.6	195.6 ± 91.8	237.2 ± 77.0	n.s.	n.s.	n.s.	n.s.
Step time (s)	0.62 ± 0.12	0.61 ± 0.11	0.59 ± 0.10	0.58 ± 0.11	0.60 ± 0.10	0.57 ± 0.08	n.s.	n.s.	n.s.	n.s.
**CV**										
Stride length	0.14 ± 0.12	0.13 ± 0.09	0.20 ± 0.20	0.17 ± 0.13	0.22 ± 0.16	0.16 ± 0.12	**0.01**	n.s.	0.08	n.s.
PAV	0.12 ± 0.09	0.10 ± 0.05	0.14 ± 0.09	0.13 ± 0.07	0.16 ± 0.10	0.12 ± 0.04	n.s.	0.09	n.s.	0.94
Step time	0.08 ± 0.04	0.08 ± 0.04	0.09 ± 0.05	0.10 ± 0.08	0.11 ± 0.08	0.10 ± 0.05	**0.02**	**0.03**	0.43	0.99

*PAV, peak shank angular velocity; STN, subthalamic nucleus deep brain stimulation; SNr, substantia nigra deep brain stimulation; ll, leg length; D, dominant; ND, non-dominant; CV, coefficient of variation*.

*p-Values are computed with Friedman test and if significant, with Wilcoxon signed rank test*.

*Bold font was decided on p < 0.05*.

**Figure 2:**
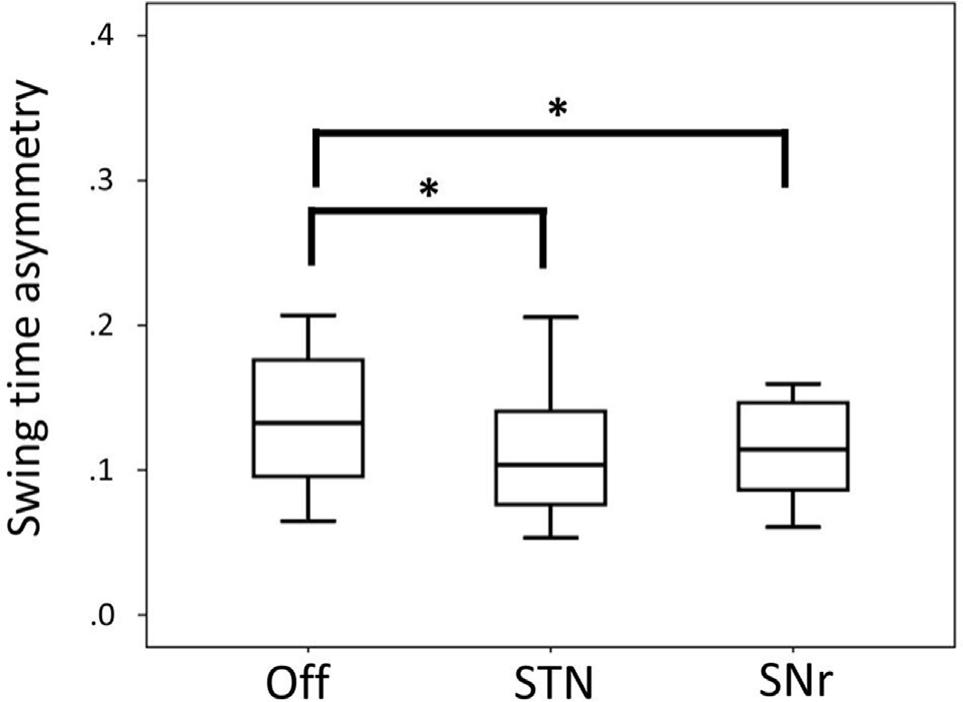
Boxplot representing median values, 25–75% range (box) and min–max range (bars) of swing time asymmetry. Differences were computed with the Wilcoxon signed rank test and are denoted by horizontal square brackets. Abbreviations: STN, subthalamic nucleus deep brain stimulation; SNr, substantia nigra deep brain stimulation; ll, leg length; CV, coefficient of variation.

Swing time asymmetry was improved by both STN and SNr stimulation. SNr stimulation also improved swing time asymmetry compared to Off stimulation (Figure 2) at *p* < 0.05 (after exclusion of PD11 two-tailed *p* = 0.062).

### Anatomical Position of SNr Contact

**Figure 3:**
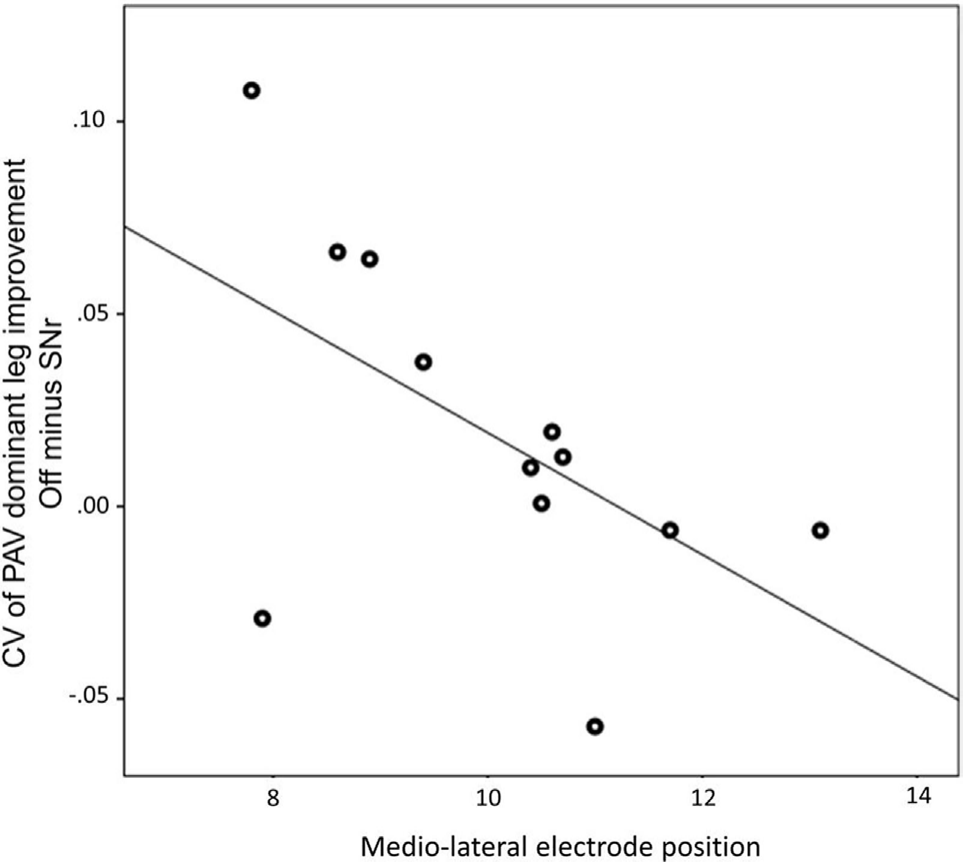
Medio-lateral location of electrode of the most caudal contact is associated with the improvement of peak angular velocity variability by substantia nigra pars reticulate (SNr) stimulation. Both electrode position and CV of PAV are obtained from the dominant side. A more medial electrode position is associated with more regular gait induced by SNr stimulation (*r* = −0.594; *p* = 0.042). Abbreviations: CV, coefficient of variation; PAV, peak shank angular velocity.

We found a negative correlation between the alteration of peak angular velocity variability of the dominant leg by SNr stimulation and the laterality of the electrodes caudal contact (*r* = −0.594, *p* = 0.042, Figure 3). This indicates that patients with a more medial electrode position show a more regular gait pattern induced by SNr stimulation. This correlation was still present after exclusion of PD11 (*r* = −0.651, *p* = 0.030). No correlation was found between electrode laterality and other gait parameters.

## Discussion

Here, we found that STN stimulation improved both spatial and temporal characteristics of gait with a stride length increment, reduction of both stride length and step time variability, and increased swing time symmetry. Furthermore, mono SNr stimulation did not modulate spatial measures, but may affect the modulation of temporal gait integration by increasing the swing time symmetry. In addition, we found that patients with a more medial electrode position of the caudal contact associated with an improvement of the gait patterns regularity induced by mono SNr stimulation.

In line with our hypotheses, SNr stimulation can modulate the temporal characteristics of the gait cycle by reducing the STA. From animal experiments, we know that the MLR and the reticulospinal tract are involved in the temporal modulation of the gait pattern. One of the target areas of the SNr is the MLR, which is considered the main locomotor center with a GABAergic connection between the SNr and the ponto-mesencephalic tegmentum (21). Our results suggest that SNr stimulation regulates temporal gait integration, presumably by modulating MLR, which has a predominant role in temporal adaptation. The PPN, as part of the MLR, can modify the cadence but not step length, as is suggested with PPN stimulation in PD patients (30). Furthermore, an MRI study revealed that increasing the gait speed during imaginary walking activates the MLR region (31). An earlier experimental clinical study on PD patients showed that SNr stimulation could improve the braking mechanism during gait initiation, but not the spatial parameters of gait (32). This decreased vertical acceleration, as well as an increased gait asymmetry, is characteristic for axial motor impairment (33). The consistence of these kinematic findings with results from PPN stimulation may support the view that nigral stimulation might entrain pedunculopontine locomotor integration (30, 31, 34).

Our results support the hypothesis that mono SNr stimulation may support regulation of temporal gait characteristics. Furthermore, patients with a more medial location of the active SNr contact acquire a more regularized gait pattern with SNr stimulation. This correlation aligns with previous findings of animal experiments showing that stimulation of the medial SNr could modulate the temporal integration of gait, as opposed to the lateral part of the SNr (21, 22).

In daily life, FOG is still an unmet therapeutic need. As reprogramming strategy, we suggested combined STN + SNr stimulation. However, the mechanism on how combined STN + SNr stimulation might reduce FOG remains unknown. We did not consider the combined STN + SNr stimulation in this study for two major reasons. First, we aimed to achieve distinct effects on locomotor variables from potentially separate STN or SNr contributions to gait integration since our primary interest was to obtain differentiated pathophysiological insight on the role of a distinct neuroanatomic target in locomotor integration. Furthermore, we were limited in the number of experimental conditions that patients under medication-off conditions could tolerate and, therefore, decided not to include a fourth condition on STN + SNr. We set the washout period between the conditions to 20 min in order to limit potential carry-over effects. Nevertheless, we cannot fully exclude carry-over effects, although a period of 20 min is considered sufficient in the advanced disease stage (25). In this sense, we excluded three patients showing no clinical difference between Off stimulation and STN stimulation, either owing to incomplete washout of STN-DBS or stimulation effect.

Another methodological consideration in this study refers to our choice to use different stimulation intensities of mono STN and mono SNr stimulation. We decided in this way, since we wished to use stimulation intensities that closely adhere to current reprogramming standards from previous reports and randomized clinical trials, including the ongoing multicenter trial (ClinTrials.gov: NCT02588) (20). We cannot fully exclude that stimulation of a dorsolateral STN contact would potentially affect adjacent structures owing to potential current spreading to adjacent structures, in particular when higher stimulation amplitudes are applied on the level of STN. Moreover, stimulation of the STN might not be limited to changing local STN activity, but might modulate adjacent structures through connectivity. The same applies for stimulation of the SNr although stimulated with lower amplitudes. On the other hand, the stimulation intensity of mono SNr stimulation was lower than that of mono STN stimulation, which could potentially account for a lower effect of SNr stimulation on clinical outcome measures. Nevertheless, higher stimulation intensities of the most caudal contact would have led to capsular side effects, in particular in more laterally localized electrodes and would have largely prevented adjustment of stimulation intensities of subthalamic and nigral contacts. Finally, the applied SNr stimulation intensities were similar to effective parameters reported in previous work (20). Only in one patient (PD11), we were limited in increasing the nigral stimulation amplitudes owing to side effects in terms of blurred vision and therefore used the highest tolerable amplitudes at 0.3 V bilaterally. As this might potentially be subthreshold for inducing therapeutic effects from nigral neuromodulation, we reevaluated our statistical findings after exclusion of PD11. This led to largely consistent findings.

The findings from this study provide closer insight into the mechanisms of STN and SNr stimulations. We suggest as major finding from this study that STN stimulation modulates both spatial and temporal measures. Instead, nigral stimulation did not modulate spatial measures, but may affect the modulation of temporal gait integration. This could be mediated through MLR modulation when our findings are interpreted in the context of established knowledge about temporal integration pathways. Furthermore, patients with a more medial position of the caudal contact of the electrode show a more regular gait pattern induced by mono SNr stimulation. The findings presented here help to decipher the enigmatic pathophysiological networks involved in gait in PD patients with FOG and encourage further studies on locomotor network modulation on the level of STN and SNr.

## Ethics Statement

This study was carried out in accordance with the recommendations of “§ 15 der Berufsordnung für Ärzte in Baden-Württemberg,” “Ethik-Kommission am Universitätsklinikum Tübingen” with written informed consent from all subjects. All subjects gave written informed consent in accordance with the Declaration of Helsinki. The protocol was approved by the “Ethik-Kommission am Universitätsklinikum Tübingen.”

## Author Contributions

MS, JK, CP, RK, AG, and DW: conception and design of the study. MS, JK, and MH: acquisition of data. MS, FB, BB, and DW: analysis and interpretation of data. MS and DW: drafting the article. All authors: revising it critically for important intellectual content. All authors: final approval of the version to be submitted.

## Conflict of Interest Statement

MS, BB, CP, JK, MH, FB, and AG declare no competing financial interest. DW received research grants from the German Research Council (DFG, WE5375/1-1, WE5375/1-3) and research funding from Medtronic, as well as speakers honoraria/travel grants from Medtronic, Abott (St. Jude), Boston Scientific, and Abbvie. RK has received research grants of the German Research Council (DFG; KR2119/8-1), the Michael J Fox Foundation, and the Fonds National de Recherche Luxembourg (FNR; PEARL and NCER-PD) as well as speakers honoraria and/or travel grants from Teva, UCB Pharma, Abbvie, Novartis, St. Jude, and Medtronic.
